# Distinct pro-vigilant profile induced in rats by the mGluR5 potentiator LSN2814617

**DOI:** 10.1007/s00213-015-3936-8

**Published:** 2015-04-24

**Authors:** Sally Loomis, Andrew McCarthy, Christopher Baxter, Daniel O. Kellett, Dale M. Edgar, Mark Tricklebank, Gary Gilmour

**Affiliations:** Lilly Research Laboratories, Eli Lilly & Co. Ltd, Erl Wood Manor, Sunninghill Road, Windlesham, Surrey, GU20 6PH England UK

**Keywords:** Vigilance, EEG, RAT, mGlu5, Potentiator, Wake, Translation, Metabotropic, Modafinil, Amphetamine

## Abstract

While treatment options are available, excessive daytime sleepiness (EDS) remains a significant unmet medical need for many patients. Relatively little rodent behavioural pharmacology has been conducted in this context to assess potential pro-vigilant compounds for their ability to restore functional capacity following experimentally induced sleep loss. Male Wistar rats were prepared for electroencephalographic (EEG) recording and subject to 11 h of sleep restriction using a biofeedback-induced cage rotation protocol. A simple response latency task (SRLT) was used to behaviourally index sleep restriction and the effects of pro-vigilant compounds: modafinil, d-amphetamine, caffeine, and the mGlu5-positive allosteric modulator LSN2814617. Sleep restriction resulted in a consistent, quantified loss of non-rapid eye movement (NREM) and REM sleep that impaired SRLT performance in a manner suggestive of progressive task disengagement. In terms of EEG parameters, all compounds induced wakefulness. Amphetamine treatment further decreased SRLT performance capacity, whereas the other three compounds decreased omissions and allowed animals to re-engage in the task. Caffeine and modafinil also significantly increased premature responses during this period, an effect not observed for LSN2814617. While all compounds caused compensatory sleep responses, the magnitude of compensation observed for LSN2814617 was much smaller than would be predicted to result from the prolongation of wakefulness exhibited. Using simple response latencies to index performance, an mGlu5 PAM dramatically increased wakefulness and improved functional capacity of sleep-restricted animals, without eliciting a proportionate compensatory sleep response. This effect was qualitatively distinct from that of amphetamine, caffeine and modafinil.

**Electronic supplementary material:**

The online version of this article (doi:10.1007/s00213-015-3936-8) contains supplementary material, which is available to authorized users.

## Introduction

Sleep deprivation and the cognitive sequelae of excessive daytime sleepiness (EDS) are prevalent in modern society. EDS presents in insomnia, sleep apnea and narcolepsy (Roth and Roehrs 1996) and often also as a complication of other neuropsychiatric disorders, such as Parkinson’s disease, multiple sclerosis, chronic pain and depression (e.g. see reviews by Boulos and Murray 2010; Chellappa et al. 2009; Knie et al. 2011). Whilst recovery sleep is the best known remedy for EDS, this is often unattainable, especially for patients exhibiting comorbidity. Unmet clinical need remains for pharmacotherapies that can restore cognitive deficits during EDS, ideally lacking potential for causing debilitating rebound hypersomnolence.

Beyond the well-established effects of caffeine, amphetamine and modafinil, several studies have already investigated novel pharmacological countermeasures for cognitive impairment caused by extended wakefulness in animals, for example AMPAkines (Porrino et al. 2005), histamine H_3_ receptor antagonists (Stocking and Letavic 2008), serotonin 5HT_6_ receptor agonists (Ly et al. 2013) and orexin receptor agonists (Deadwyler et al. 2007). However, little preclinical behavioural work so far has focused on sleep-restricted rodents or explored the utility of simple response latency testing in the rat as a translational homologue of the human psychomotor vigilance task (PVT) for detection of pro-vigilant pharmacological effects. Aspects of attentional performance are typically the first and most sensitive processes to be negatively affected by sleep loss/EDS (Balkin et al. 2004). This is of significant functional consequence as deficits in sustained or vigilant attention are likely to be the root cause of sleep loss-related accidents (Akerstedt et al. 2011), and it is easy to hypothesize how attentional deficits could underlie or catalyze other types of cognitive deficit in this context. The PVT was developed to measure exactly this type of vigilant or sustained attention in human studies of sleep loss and performance capacity (Balkin et al. 2004; Dinges and Powell 1985). It tests simple reaction times (RTs) to a visual cue occurring at variable inter-stimulus intervals over a short period. The PVT is known to be sensitive to both circadian and homeostatic sleep drives (Van Dongen and Dinges 2000). Sleep deprivation causes a general slowing of RTs in PVT, where an especially sensitive measure in humans is the worsening of slower RTs: >500 ms latencies known as performance “lapses” (Anderson et al. 2010; Basner and Dinges 2011). However, as well as performance lapses, increased errors of omission and commission, and an enhancement of time-on-task detriments are all evident deprivation-related deficits (Lim and Dinges 2008).

Previous reports have highlighted the potential of metabotropic glutamate receptor 5 (mGlu5) positive allosteric modulators (PAMs) to mediate wake-promoting effects of apparently large magnitude in rats (Gilmour et al. 2013; Gregory et al. 2013). However, it is not clear from the previous work, which focused assessment only on EEG-sleep/wake state parameters in rested animals, whether mGlu5 PAM-induced wakefulness extends to states of sleep restriction, and further whether it is functionally beneficial. The aim of this paper therefore was to directly compare an mGlu5 PAM, namely LSN2814617, to the known wake-promoting agents modafinil, caffeine and amphetamine on the performance of a simple response latency task (SRLT) in the rat following biofeedback-induced sleep restriction.

## Methods

### Biofeedback-induced sleep restriction

All experimental protocols were approved by the local ethics committee and carried out in accordance with the UK Animals (Scientific Procedures) Act 1986. Adult, male Wistar rats (approximately 270–300 g at time of surgery, Charles River Laboratories, Margate, UK) were prepared with cranial implants for chronic electroencephalogram (EEG) and electromyogram (EMG) recording (Supplement S1). Following SRLT training as described below, animals were housed individually in custom-designed, sleep deprivation chambers for the duration of the experiment. Each chamber consisted of a cylinder (39.7 cm diameter by 32.1 cm length) constructed of plexiglass rods, positioned horizontally inside a Plexiglas frame (637.2 cm^2^ floor space). The study was conducted in a sound-attenuated, light and temperature-controlled recording room, to control for sensory modalities known to affect sleep. The cranial implants were connected to ultra-low-torque slip-ring commutators (Hypnion, Inc., Lexington, MA, USA) by metal coil reinforced flexible cables, allowing for free, unrestrained movement throughout the cage. When an epoch of NREM or REM sleep was detected, the program (*SCORE*-2004™; see Supplement S1) activated a motor to rotate the cylindrical chamber around its axis for 10 s at a rate of 18.3 cm/s. This gives a mild vestibular stimulus sufficient to cause immediate awakening and a small amount of locomotion during the short period of rotation. The direction in which the chamber rotated was pseudorandomly determined each time. Each 10 s epoch in which a rotation occurred reflected an attempt to enter NREM or REM sleep.

### Simple response latency task

SRLT testing was conducted in standard operant boxes housed in sound and light attenuation chambers (Med Associates, US). Food pellets (Noyes, 45 mg, Formula P) were delivered from an automatic pellet dispenser. The houselight and magazine light provided preparatory and imperative cues, respectively. Experimental sessions were controlled and data recorded by programs written in-house using MedPC IV software (Med Associates, UK). Operant boxes were equipped with dummy commutators so that EEG implant tethers could be connected to prevent them from disturbing the implanted animals during testing.

### Training

**Fig. 1:**
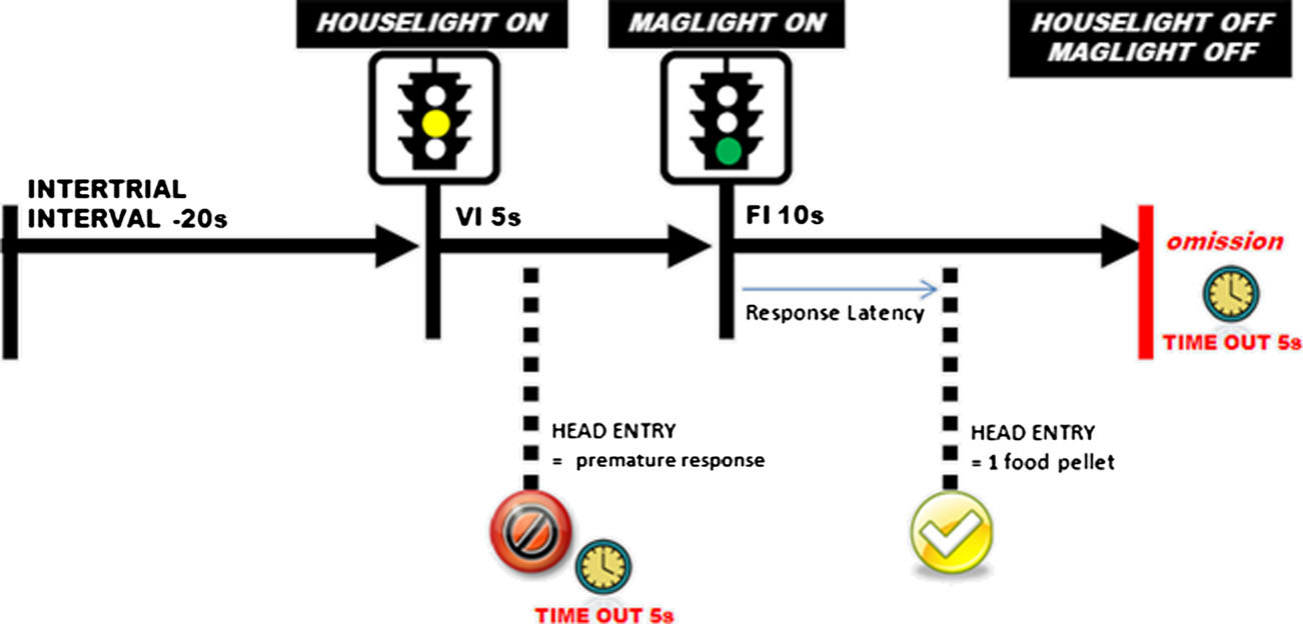
Task overview. Following an inter-trial interval (20 s), the houselight is illuminated to serve as a preparatory cue. After a 5-s variable interval (range 4–6 s), the magazine light is illuminated to serve as an imperative cue. A head entry made during the 10 s period when the maglight is on will result in the delivery of a food pellet. Head entries made before magazine light onset are recorded as premature responses, and a failure to respond to the magazine light is recorded as an omission. Both premature responses and omissions are “punished” with a 5-s time out period

Following recovery from surgery rats were maintained on food restriction until they reached the final training programme and then returned to ad libitum food. Animals were trained on the task in a series of successive approximations during daily 30 min sessions. In the first stage, the magazine light/imperative cue was illuminated for 10 s (with a 30 s inter-trial interval) during which a nosepoke would earn a food pellet reward. Food reward would be delivered at the end of this cue even if no nosepoke was made. The houselight/preparatory cue remained illuminated throughout the session except for 5-s timeout periods that occurred while the animal collected food rewards. Animals were required to make at least ten nosepokes to advance to the second stage, where all rewards had to be obtained by nosepoke. If the animal did not make a nosepoke during the presentation of the imperative cue, an omission was recorded that resulted in a 5-s timeout period with no light stimulus. After completing >75% of trials available over two consecutive sessions, animals could progress to the third stage where they had to learn to inhibit premature responding during the preparatory cue. Thus, a trial would be initiated by illumination of the houselight and responding had to be inhibited until illumination of the magazine light. During this stage, the interval between preparatory and imperative cue was fixed at 5 s. Premature responses during the preparatory cue resulted in a timeout period. Again, the criterion for progression to the next stage of training was completion of >75% of trials available over two consecutive sessions. Where necessary, animals finding it difficult to meet this criterion would be subjected to remedial training using shorter fixed intervals between cues. In the final stage of training, a variable interval (range 4–6 s) was introduced between preparatory and imperative cues to prevent animals from timing responses precisely. From the SRLT, the number of trials completed, head entries made and response errors (premature responses and omissions) were counted, RTs, premature response latencies and the times at which both occurred during the session were also collected. SRLT testing protocol is illustrated in Fig. 1.

### Drugs

d-amphetamine sulfate (Sigma Aldrich, UK) was dissolved in 5 % (*w/v*) glucose solution and administered subcutaneously at a volume of 1 ml/kg, in doses of 0.5, 1 and 2 mg/kg. Modafinil (Apin Chemicals UK) was dissolved in 0.25 % methylcellulose (15 cP) and administered intraperitoneally at a volume of 2 ml/kg, in doses of 30, 100 and 300 mg/kg. LSN2814617 [(7S)-3-tert-butyl-7-[3-(4-fluorophenyl)-1,2,4-oxadiazol-5-yl]-5,6,7,8-tetrahydro[1,2,4]triazolo[4,3-A]pyridine (Lilly Research Labs)] was suspended in 1 % (*w/v*) carboxymethyl cellulose, complemented with addition of 0.25 % Tween 80 and 0.05 % antifoam. It was administered orally at a volume of 1 ml/kg, in doses of 1, 3 and 10 mg/kg. Caffeine (Lilly Research Labs) was dissolved in 0.25 % methylcellulose (15 cP) and administered intraperitoneally at a volume of 2 ml/kg, in doses of 4, 8 and 12 mg/kg. All doses refer to free base or acid weights of compounds.

### Study design

Sleep restriction protocols were run in a within-subjects crossover study design, where rats were pseudo-randomly assigned using a Latin square to one drug treatment condition per week. Each compound was tested in a separate experiment, and between experiments new animals were used (*n* = 95). Both a 300 mg/kg treatment of modafinil and a 3 mg/kg treatment of LSN 2814617 acted as positive controls to enable bridging between experiments. For the final analysis, modafinil and LSN 2814617 treatment groups were combined across studies resulting in larger sample sizes for these drugs.

**Fig. 2:**
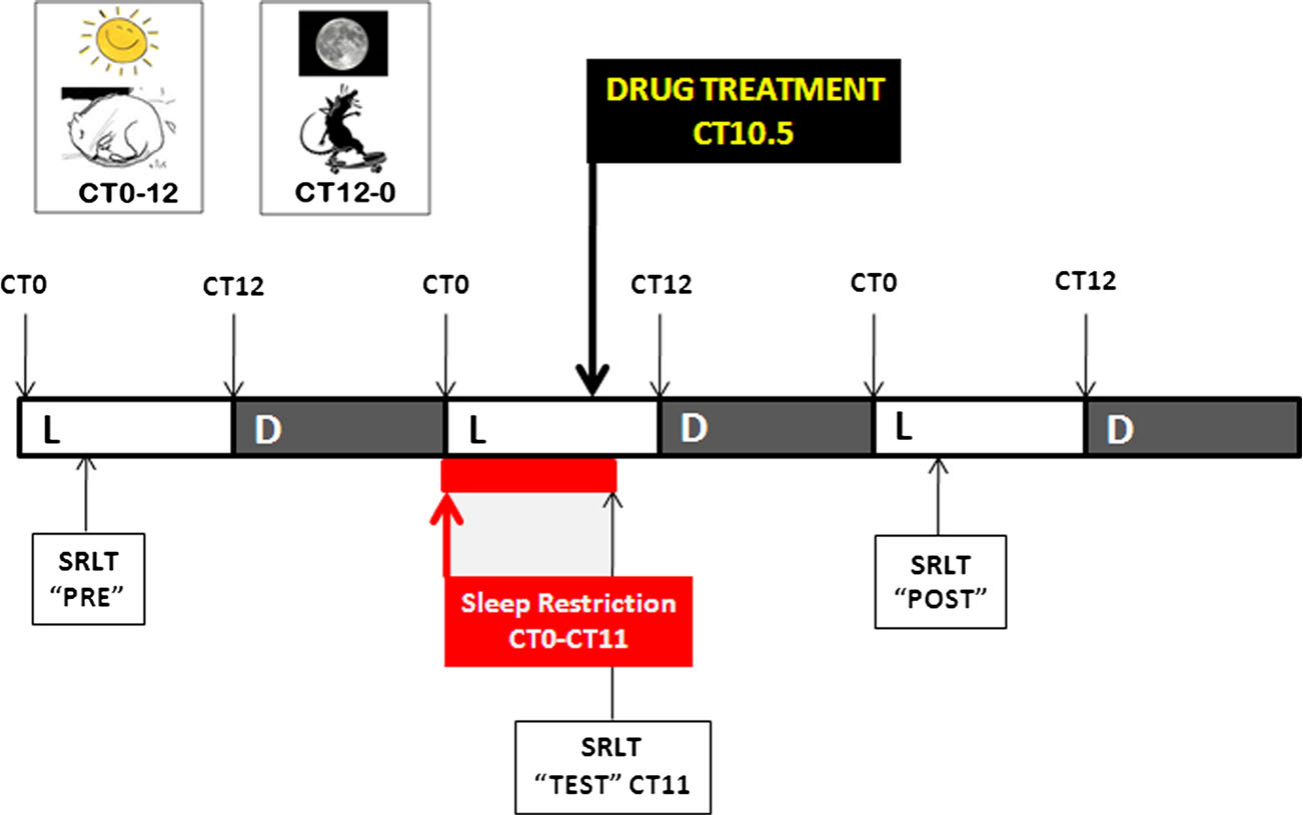
Study design. Rats were kept on a Light (L) phase–Dark (D) phase 12:12 cycle (8.00 a.m–8.00 p.m.). For baseline data, rats underwent simple response latency testing at Circadian Time (CT) 3–5 on “Pre” day. On “Test” day sleep deprivation commenced at onset of Light Phase and was continued for 11 h (CT0-CT11). All drugs were dosed at CT10.5 (10.5 h after lights on). At CT11 animals were removed from sleep deprivation wheels and placed immediately into operant boxes for a 40-min SRLT session. Rats were then retested in the SRLT on “Post” day between CT 3–5

To administer a drug treatment, rats were removed from their cage for about 60–90 s to be dosed. At least 7 days “washout” was allowed preceding and following treatments with no duplication of treatment within an animal. Prior to each study, all subjects underwent a 5-h sleep restriction process to habituate animals to the procedure. All drugs were dosed at circadian time 10.5 (CT-10.5; 10.5 h after lights on, LD 12:12). For 3 consecutive days (“Pre”, “Test” and “Post”), animals were removed from sleep deprivation wheels and placed immediately into operant boxes for a 40-min SRLT session. On “Test” day, SRLT testing occurred immediately following an 11-h sleep restriction period from CT0 to CT11. On “Pre” and “Post” days, rats underwent SRLT testing between CT3 and CT5. Study design is depicted in Fig. 2. For REM and NREM sleep parameters, differential measures were calculated between baseline “Pre” day phase data and four other sections of “Test” and “Post” day phase data: “Sleep Deprivation” = Test Day Light Phase–Pre Day Light Phase, indexing the direct effect of the sleep restriction process relative to a normal resting phase; “Drug Treatment” = Test Day Dark Phase–Pre Day Dark Phase, indexing the effect of drug treatment during the post-sleep restriction waking phase relative to a normal waking phase; “Recovery – Light” = Post Day Light Phase–Pre Day Light Phase, indexing the potential for recovery sleep following sleep restriction and drug treatment in the subsequent light phase by comparing the “Post” day resting phase relative to a normal resting phase; “Recovery – Dark” = Post Day Dark Phase–Pre Day Dark Phase, indexing the potential for recovery sleep following sleep restriction and drug treatment in the subsequent dark phase by comparing the “Post” day waking phase relative to a normal waking phase. Following exclusion of datasets that were subject to technical error, each drug study comprised of the following sample sizes: modafinil, *n* = 21; d-amphetamine, *n* = 13; caffeine, *n* = 11; LSN2814617, *n* = 23. No other exclusion criteria were applied to datasets.

### Statistics

All data were analyzed using Statistica v9.0 (Statsoft Ltd, Bedford, UK). For general sleep/wake state parameter assessments, the first 5 h following drug administration were analyzed with repeated measures ANOVA, with “Treatment” and “Time” as within-subjects factors. Planned comparisons of each treatment group compared to the vehicle group were also conducted at each time point for each drug. SRLT task parameters were analyzed with repeated measures ANOVA, with “Treatment” and “Day” as within-subjects factors. Planned comparisons were then conducted, where respective vehicle- and drug-treated groups were compared between Pre Day–Test Day and Pre Day–Post Day, and also for each separate day a planned comparison was made between the vehicle group and the drug-treated group. Drug effects on REM and NREM sleep differential measures were analysed using. ANOVAs with “Treatment” as a within-subject factor.

## Results

Full details of results of statistical analyses calculated for all datasets can be found as Electronic Supplementary Material (Supplement S2).

### General sleep/wake parameters

**Fig. 3:**
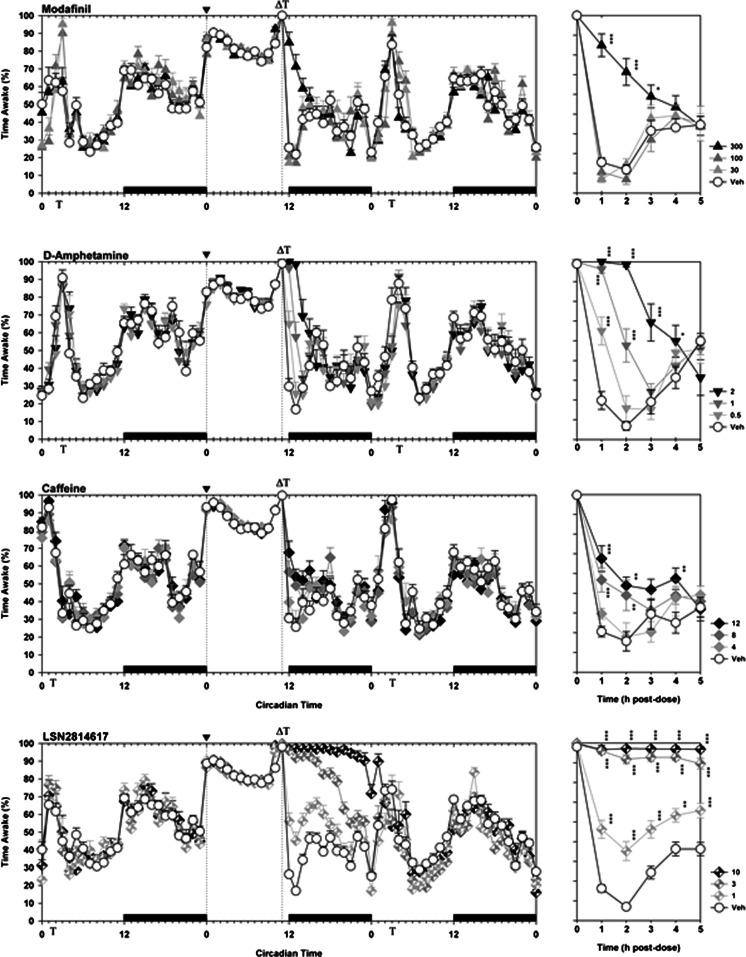
The effect of pro-vigilant compounds on wakefulness in rats. The graphs on the left depict a 72 h plot of wakefulness for modafinil, d-amphetamine, caffeine and LSN2814617 studies, thereby including “Pre”, “Test” and “Post” drug administration days. The *x*-axis depicts Circadian Time, where the solid bars above the bottom *x*-axis between CT12-0 indicate when dark phases occurred. *Vertical dotted lines* at CT0 and CT11 depict the beginning and end of the sleep restriction period, where it can be clearly seen that sleep restriction results in marked wakefulness during this period compared to the “Pre” day. The beginning of the sleep restriction period is also marked by the symbol “▼” on the top *x*-axis. The symbol “**Т**” on the top and bottom *x*-axes indicates when SRLT testing occurred each day, and the symbol “**Δ”** on the top *x*-axis indicates when drugs were administered. The graphs on the right are a zoomed view of the first 5 h of wakefulness following drug administration. *Asterisks* refer to planned comparisons of drug treatment groups against the respective Veh group for each study. *p < 0.05; **p < 0.01; ***p < 0.001

A baseline period of 24 h was used to evaluate the sleep history of individual animals prior to treatment. During this time, all rats displayed a circadian rhythm in sleep, activity and body temperature that was consistent between treatment groups. As depicted in Fig. 3, in the face of 11 h of sleep restriction, all four compounds significantly increased wakefulness for several hours following dosing (Main Effects of Drug: modafinil F_3,54_ = 20.8, *p* < 0.0001; amphetamine F_3,40_ = 29.8, *p* < 0.0001; caffeine F_3,35_ = 11.3, *p* < 0.0001; LSN2814617 F_3,73_ = 456.6, *p* < 0.0001). Increases in wakefulness caused by d-amphetamine, caffeine and LSN2814617 were dose dependent, although only the highest dose (300 mg/kg) of modafinil induced any significant effects. Wake-promoting effects of modafinil, amphetamine and caffeine had effectively dissipated by 3–5 h post-dose, while in contrast LSN2814617 effects were larger in magnitude and more persistent compared to the other compounds. The highest dose of LSN2814617 tested (10 mg/kg) induced a near 100% wakefulness throughout the subsequent 12 h dark phase.

### SRLT functional effects

**Fig. 4:**
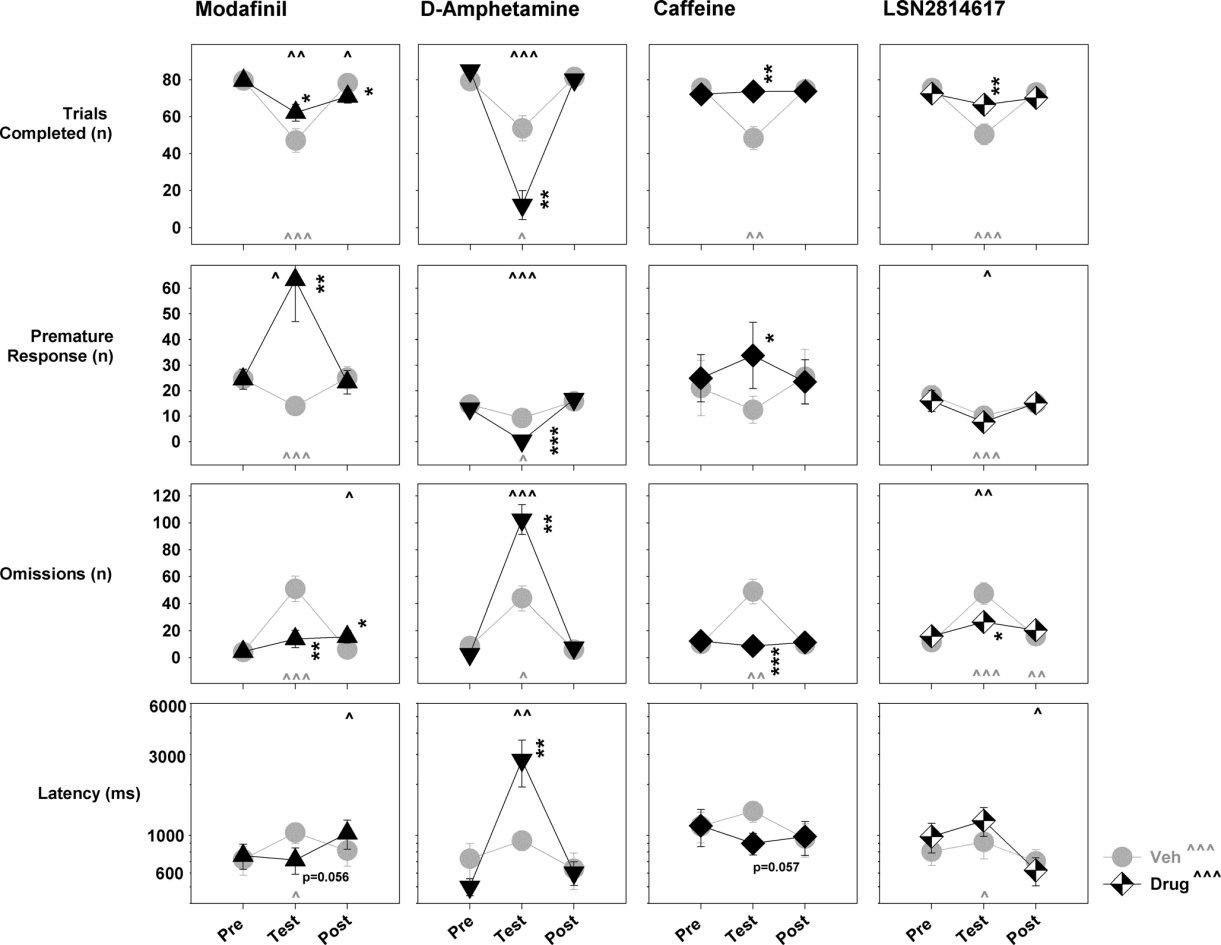
Pro-vigilant drug effects on SRLT response parameters in 11-h sleep-restricted rats. The four main SRLT response parameters are depicted from top to bottom in the following order: number of trials completed, number of premature responses, number of omissions and response latencies. The doses of drugs depicted were 300 mg/kg modafinil, 1 mg/kg amphetamine, 12 mg/kg caffeine and 3 mg/kg LSN2814617. For individual graphs, the *x*-axis depicts the summary of each measure for the “Pre”, “Test” and “Post” drug administration test session. *Asterisks* refer to planned comparisons of Veh versus Drug treatment within a test session for each study, where *p < 0.05; **p < 0.01; ***p < 0.001. Carets refer to planned comparisons for either Veh (*light grey*) or Drug (*black*) treatments between test sessions, where **^** *p* < 0.05; **^^** *p* < 0.01; **^^^** *p* < 0.001, in comparison to the “Pre” session.

The effects of sleep restriction and drug administration on the simple response latency task are depicted in Fig. 4. A qualitatively consistent effect of sleep restriction was present across all four drug studies, whereby a marked, significant decrease in completed trials (vehicle group, planned comparisons of “Pre” day to “Test” day performance, modafinil *p* < 0.0001; amphetamine *p* = 0.013; caffeine *p* = 0.002; LSN2814617 *p* < 0.0001) and increase in omissions (vehicle group, planned comparisons of “Pre” day to “Test” day performance, modafinil *p* < 0.0001; amphetamine *p* = 0.01; caffeine *p* = 0.001; LSN2814617 *p* < 0.0001) was evident. Sleep restriction significantly decreased premature responding in three out of four studies (vehicle group, planned comparisons of “Pre” day to “Test” day, modafinil *p* = 0.001; amphetamine *p* = 0.012; LSN2814617 *p* = 0.001), while also showing a nominal decrease in the caffeine study. Response latencies were significantly increased in the modafinil and LSN2814617 studies by sleep restriction (vehicle group, planned comparisons of “Pre” day to “Test” day, modafinil *p* = 0.023; LSN2814617 *p* = 0.012), but not in the amphetamine and caffeine studies. With the exception of an increase in omissions during the LSN2814617 study, no significant carry-over effects of sleep restriction could be detected on any SRLT parameter during the “Post” test session.

When drug doses were chosen for comparison on the basis of pragmatically equivalent pro-vigilant effects on EEG parameters (300 mg/kg modafinil, 1 mg/kg amphetamine, 12 mg/kg caffeine, and 3 mg/kg LSN2814617), their effects on sleep restriction-impaired task performance were markedly different. Modafinil, caffeine and LSN2814617 all significantly normalized sleep restriction-impaired performance with regard to both trials completed (“Test” day, planned comparisons of “Veh” to “Drug” group performance, modafinil *p* = 0.046; caffeine *p* = 0.005; LSN2814617 *p* = 0.014) and omissions (“Test” day, planned comparisons of “Veh” to “Drug” group performance, modafinil *p* = 0.002; caffeine *p* = 0.001; LSN2814617 *p* = 0.018) measures. Both caffeine and modafinil significantly increased premature responding (“Test” day, planned comparisons of “Veh” to “Drug” group performance, modafinil *p* = 0.005; caffeine *p* = 0.034) following dosing after sleep restriction. The effect of modafinil was particularly marked for this parameter, where premature response rate more than doubled during the “Test” session. LSN2814617 did not significantly affect premature response rates following administration. With regard to response latency, both caffeine and modafinil displayed a non-significant trend towards decreasing this measure on test day (“Test” day, planned comparisons of “Veh” to “Drug” group performance, modafinil *p* = 0.058; caffeine *p* = 0.057), while LSN2814617 was without effect. In dramatic comparison to these three compounds, all of which showed some evidence of functionally improving performance capacity following sleep restriction, animals dosed with d-amphetamine could not complete the SRLT task properly. On “Test” day, d-amphetamine-treated animals displayed a further marked and significant decrease in completed trials (“Test” day, planned comparisons of “Veh” to “Drug” group performance, amphetamine *p* = 0.008), increase in omissions (“Test” day, planned comparisons of “Veh” to “Drug” group performance, amphetamine *p* = 0.008) and a dramatic increase in response latencies (“Test” day, planned comparisons of “Veh” to “Drug” group performance, amphetamine *p* = 0.008). Finally, in terms of a “Post” session testing impairment, only modafinil displayed a small but significant decrease in trials completed (“Test” day, planned comparisons of “Veh” to “Drug” group performance, modafinil *p* = 0.027) and increase in omissions (“Test” day, planned comparisons of “Veh” to “Drug” group performance, modafinil *p* = 0.041) and response latency on the day following dosing.

### Sleep parameters and compensatory sleep response effects.

**Fig. 5:**
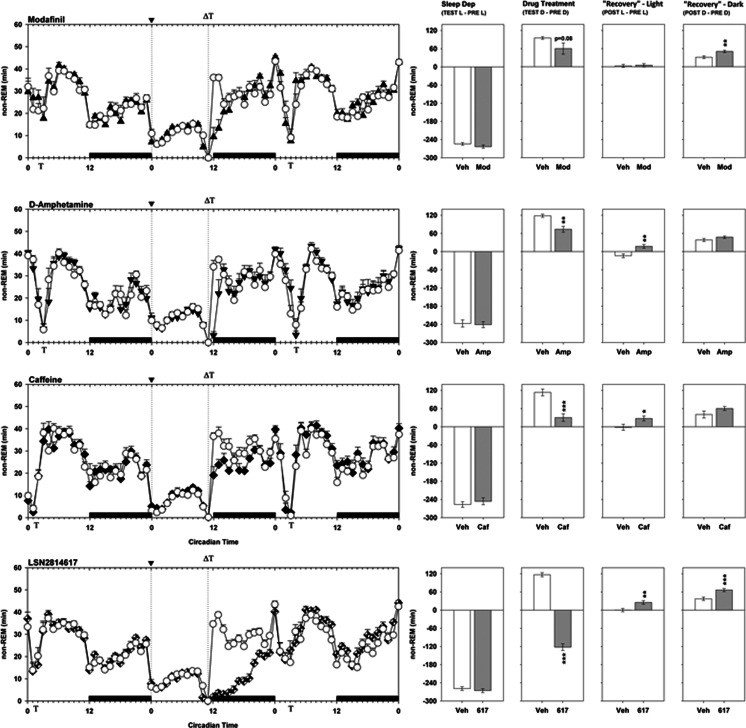
The effect of pro-vigilant compounds on non-REM sleep parameters in 11 h sleep-restricted rats. The graphs on the left depict a 72 h plot of time spent in non-REM sleep for modafinil (300 mg/kg), d-amphetamine (1 mg/kg), caffeine (12 mg/kg) and LSN2814617 (3 mg/kg) studies, thereby including “Pre”, “Test” and “Post” drug administration days. The *x*-axis depicts Circadian Time, where the solid bars above the bottom *x*-axis between CT12-0 indicate when dark phases occurred. *Vertical dotted lines* at CT0 and CT11 depict the beginning and end of the sleep restriction period, where it can be clearly seen that sleep restriction results in marked wakefulness during this period compared to the “Pre” day. The beginning of the sleep restriction period is also marked by the symbol “▼” on the top *x*-axis. The symbol “**Т”** on the top and bottom *x*-axes indicates when SRLT testing occurred each day, and the symbol “**Δ”** on the top *x*-axis indicates when drugs were administered. The graphs on the right are summary measures of the differential amounts of REM sleep achieved by rats between different circadian phases of the study. The different measures were calculated as follows: *Sleep Dep* = *“Test” Light Phase–“Pre” Light Phase*; Drug Treatment = *“Test” Dark Phase–“Pre” Dark Phase*; “Recovery” Light = *“Post” Light Phase–“Pre” Light Phase*; “Recovery” Dark = *“Post” Dark Phase–“Pre” Dark Phase. Asterisks* refer to planned comparisons of drug treatment groups against the respective Veh group for each study. *p < 0.05; **p < 0.01; ***p < 0.001

By comparison to the same time period the day before, the biofeedback method of sleep restriction caused a loss of approximately 6 h NREM sleep (Fig. 5). In the “Drug Treatment” period after sleep restriction, vehicle-treated animals recovered 90–120 min NREM sleep and 38–47 min REM sleep. Both d-amphetamine and caffeine significantly decreased the amount of NREM sleep recovered by animals during this period (amphetamine F_1,8_ = 15.7, *p* = 0.004; caffeine F_1,7_ = 36.4, *p* = 0.001), while modafinil also showed a non-significant trend towards the same effect (modafinil F_1,13_ = 3.5, *p* = 0.084). LSN2814617 had the most marked effect of all of the drugs tested, where animals significantly incurred further sleep debt, losing a further 2 h of NREM sleep during this period relative to controls (LSN2814617 F_1,16_ = 397.9, *p* < 0.0001). All drug treatment groups showed some level of NREM recovery during the subsequent day (Table 1). d-amphetamine- and caffeine-treated animals displayed a significant increase in NREM sleep during the subsequent light phase (amphetamine F_1,8_ = 14.4, *p* = 0.005; caffeine F_1,7_ = 11.2, *p* = 0.012), while for modafinil-treated animals it was delayed to the subsequent dark phase (modafinil F_1,13_ = 15.5, *p* = 0.002). LSN2814617-treated animals displayed increases in NREM sleep during both subsequent light and dark phases (“Rebound Light” F_1,16_ = 13.1, *p* = 0.002; “Rebound Dark” F_1,16_ = 23.6, *p* < 0.0001).

**Table 1:** Summary of pro-vigilant drug effects on sleep recovery parameters in sleep-restricted rats. Values presented are means and standard errors of the mean

	Vigilance state	Deficit (min)	Recovery (min)	Recovery (%)
Vehicle	Non-REM	253 ± 4	145 ± 5	57
	REM	47 ± 2	39 ± 3	82
d-Amphetamine (1 mg/kg)	Non-REM	45 ± 11	40 ± 8	89
	REM	21 ± 8	11 ± 4	53
Caffeine (12 mg/kg)	Non-REM	83 ± 14	49 ± 15	59
	REM	19 ± 8	9 ± 6	46
Modafinil (300 mg/kg)	Non-REM	35 ± 19	21 ± 7	60
	REM	15 ± 6	12 ± 5	84
LSN2814617 (3 mg/kg)	Non-REM	239 ± 12	54 ± 6	23
	REM	54 ± 5	38 ± 5	70

**Fig. 6:**
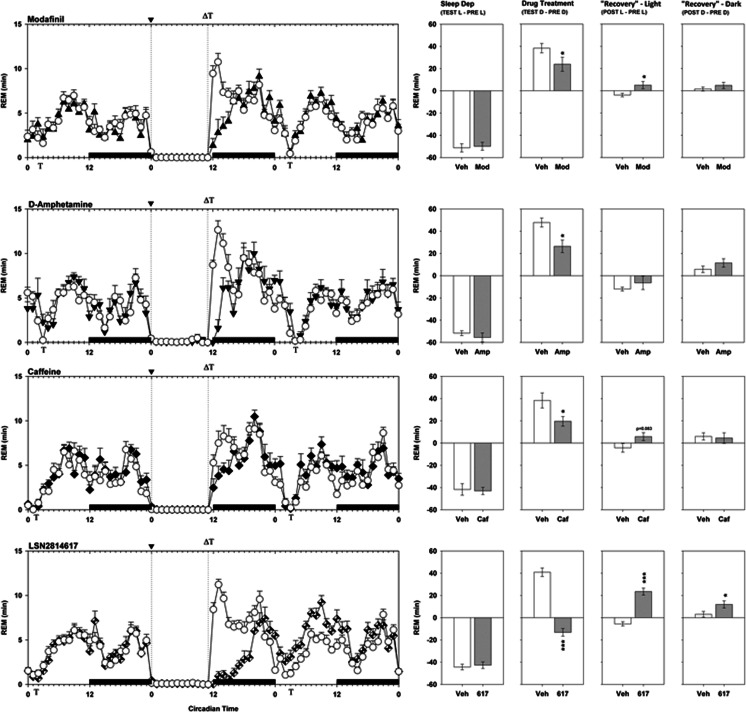
The effect of pro-vigilant compounds on REM sleep parameters in 11 h sleep-restricted rats. The graphs on the left depict a 72 h plot of time spent in REM sleep for modafinil (300 mg/kg), d-amphetamine (1 mg/kg), caffeine (12 mg/kg) and LSN2814617 (3 mg/kg) studies, thereby including “Pre”, “Test” and “Post” drug administration days. The *x*-axis depicts Circadian Time, where the *solid bars* above the bottom *x*-axis between CT12-0 indicate when dark phases occurred. *Vertical dotted lines* at CT0 and CT11 depict the beginning and end of the sleep restriction period, where it can be clearly seen that sleep restriction results in marked wakefulness during this period compared to the “Pre” day. The beginning of the sleep restriction period is also marked by the symbol “▼” on the top *x*-axis. The symbol “**Т”** on the top and bottom *x*-axes indicates when SRLT testing occurred each day, and the symbol “**Δ”** on the top *x*-axis indicates when drugs were administered. The graphs on the right are summary measures of the differential amounts of REM sleep achieved by rats between different circadian phases of the study. The different measures were calculated as follows: *Sleep Dep* = *“Test” Light Phase–“Pre” Light Phase*; *Drug Treatment* = *“Test” Dark Phase–“Pre” Dark Phase*; *“Recovery” Light* = *“Post” Light Phase–“Pre” Light Phase*; *“Recovery” Dark* = *“Post” Dark Phase–“Pre” Dark Phase*. Asterisks refer to planned comparisons of drug treatment groups against the respective Veh group for each study. *p < 0.05; **p < 0.01; ***p < 0.001

Drug effects on REM sleep profile following sleep restriction are shown in Fig. 6. All animals lost just over 40 min of REM sleep time during the sleep restriction period, which in vehicle-treated animals was almost completely recovered during the “Drug Treatment” period. All four compounds significantly decreased the amount of REM sleep recovered during this period (modafinil F_1,13_ = 5.6, *p* = 0.035; amphetamine F_1,8_ = 7.4, *p* = 0.026; caffeine F_1,7_ = 6.2, *p* = 0.042; LSN2814617 F_1,16_ = 133.7, *p* < 0.0001), while for LSN2814617 animals significantly accumulated a further 20 min of REM sleep debt. During the subsequent day, d-amphetamine and caffeine did not show significant REM compensatory sleep effects, although there was a non-significant trend for caffeine to increase REM sleep during the subsequent light phase (caffeine F_1,7_ = 4.1, *p* = 0.083). Modafinil displayed a modest but significant increase in REM sleep during the subsequent light period (modafinil F_1,13_ = 5.8, *p* = 0.031). LSN2814617 displayed the strongest REM compensatory effect, by markedly and significantly increasing REM sleep during both the subsequent light and dark phase (“Rebound Light” F_1,16_ = 71.3, *p* < 0.0001; “Rebound Dark” F_1,16_ = 6.1, *p* = 0.025).

## Discussion

By using an EEG biofeedback-induced sleep restriction methodology, reliable and quantifiable levels of sleep restriction were induced in rats that could be behaviourally indexed by a variant of the psychomotor vigilance test. The administration of exemplar pro-vigilant compounds, at doses with broadly comparable effects on EEG sleep-wake parameters, demonstrated that it was possible to determine differences in the capacity for task engagement induced by different agents. mGlu5 PAMs were identified as a novel pharmacological class of pro-vigilant drugs that induce a functionally distinct form of capacity for task engagement compared to the existing standards modafinil, amphetamine and caffeine.

The study of sleep restriction in rats has been fraught with methodological issues, which can generate confounds related to stress (Coenen and van Luijtelaar 1985; Rechtschaffen et al. 2002; Wurts and Edgar 2000) or require additional groups to control for activity/fatigue (e.g. Christie et al. 2008). In the present study, the EEG biofeedback-induced cage rotation protocol employed the minimum amount of movement necessary to wake the animal via vestibular reflex upon detection of an epoch of sleep, minimising both stress and movement confounds as best as possible. This work also benefited from quantification of EEG parameters throughout, allowing exact definition of the sleep-wake state of animals during both restriction and recovery periods. To date, very few behavioural pharmacological studies in sleep restricted rats have collected such information to guide interpretation.

When an 11 h sleep restriction was applied prior to the performance of a SRLT, rats very consistently lost around 6 h of NREM sleep and 40 min of REM sleep. This magnitude of sleep loss was sufficient to cause behavioural impairment on the SRLT task where the most marked effect was a large increase in errors of omission, accompanied by a smaller decrease in premature response rate and lengthening of RT. In humans, such effects on PVT performance can be observed after experimental total sleep deprivation or chronic sleep fragmentation (Van Dongen et al. 2003), but also as a consequence of clinically presented EDS (Czeisler et al. 2005; Dinges and Weaver 2003). A noticeable qualitative difference was evident between rat SRLT and human PVT with regard to sleep restriction effects on the RT parameter itself. These are very reliably detected in human studies, with performance lapses (i.e. RTs > 500 ms) being a defining hallmark of sleep restriction (Lim and Dinges 2008). However, during rat SRLT performance, the effects of sleep restriction on RT appeared to be relatively smaller. Future work should determine whether such discrepancies are related to motivational factors (i.e. animals responding for food versus humans adhering to verbal instruction), attentional load and/or arousal level differences.

Little preclinical work has actually studied the effect of sleep restriction on attentional processes in rodents, but the present results do display broad consistencies with the small, extant literature. For instance, it has been shown in rats that 24 h sleep deprivation (constant wheel turning method) followed by a PVT-like task (Christie et al. 2008) and 10 h total sleep deprivation (gentle handling method) followed by 5-choice serial RT testing (Cordova et al. 2006) impaired task performance. In both cases, response latencies and omissions/lapses increased significantly as a result of increased sleepiness, although the number of premature responses did not change. The induction of a change in premature response rate by sleep restriction may therefore depend on subtleties of assay protocols employed.

The most important overall finding from the present study was that different pro-vigilant pharmacologies had differential effects on the ability to restore rat SRLT performance despite all having significant effects on sleep/wake as assessed by EEG parameters. Of the four compounds tested, amphetamine (1 mg/kg) was the only drug to have marked negative effect on performance capacity of sleep-restricted animals, such that they completely disengaged from the task. Omissions increased drastically, and when animals did complete trials they occurred with very long response latencies. This profile is suggestive that this dose of amphetamine, while wake-promoting in EEG measures, results in the expression of stimulant hyperactivity in sleep-restricted rats that competes with task engagement. This represents a potentially important disconnect to existing clinical data, whereby most human studies report positive effects of standard 10 or 20 mg amphetamine doses on attentional and other cognitive tasks following sleep deprivation (see review by Minzenberg and Carter 2008). By comparison, caffeine (12 mg/kg), modafinil (300 mg/kg) and the mGlu5 PAM LSN2814617 (3 mg/kg) all had beneficial effects on SRLT performance in sleep-restricted rats. All three compounds significantly decreased omissions and increased the number of trials completed, allowing sleep-restricted animals to engage in the task more effectively. Caffeine and modafinil also displayed a trend-level tendency towards normalizing response latencies, although both compounds also had concomitant negative effects on premature response rates. Increases in premature responses were especially dramatic following modafinil administration, almost trebling in rate during the test session immediately following sleep restriction. Again, this finding is somewhat at odds with clinical data, which describes modafinil to be well tolerated with largely positive effects on human performance capacity and response inhibition parameters (Minzenberg and Carter 2008). Preclinical modafinil effects have been much more mixed, however, with reports of positive effects on stop signal (Eagle et al. 2007) and 5-choice serial RT (5CSRT) performance (Morgan et al. 2007) in normal animals, but negative effects on accuracy and impulse control in 5CSRT in normal (Waters et al. 2005) and REM sleep deprived (disc over water method) animals (Liu et al. 2011) in other studies. Several variables, including strain and age of animals, dose of modafinil, assay designs and protocol variants, may need to be carefully considered here to understand this mixture of effects.

The mGlu5 PAM LSN2814617 was found to have a remarkably powerful effect on EEG-defined wakefulness, considerably larger and more enduring than the effects observed for the other agents tested. Previous work on mGlu5 pharmacology has suggested that potentiation at this receptor can promote EEG-defined wakefulness in normal animals (Ahnaou et al. 2015; Gilmour et al. 2013; Gregory et al. 2013). Ahnaou and colleagues found that a 3 mg/kg dose of LSN2814617 enhanced slow alpha band oscillatory activity (defined as 8–11 Hz activity in their study) and reduced functional network connectivity, which the authors suggested may have been indicative of potential for impairment of cognition following dosing. In the present study, a 3 mg/kg LSN2814617 dose also increased power across an 8–11 Hz frequency band (data not presented), but by extending assessment of LSN2814617 to the measurement of functional capacity in a sleep-restricted state, the question related to the potential for cognitive impairment could also be addressed. LSN2814617 actually presented with the ability to restore functional capacity in sleep-restricted rats, displaying similar positive effects as modafinil and caffeine on trials completed and omission measures, but lacking significant effect on premature responding. Little is known at present as to why this pharmacology has such a marked effect on vigilance, although it may relate to the ability of mGlu5 receptor activation to directly and indirectly promote excitatory transmission via the NMDA receptor (Bird and Lawrence 2009). In a broader sense, the hypothesis that promotion of excitatory glutamatergic neurotransmission could be a potential mechanism of pro-vigilant effects is also substantiated by work on AMPAkine pharmacology. In this regard, the AMPA receptor PAM CX717 can restore performance of a delayed match-to-sample task in sleep deprived (gentle handling) nonhuman primates (Porrino *et al.*^2005^), whilst also normalizing sleep deprivation-induced decreases in NMDA-mediated intracellular calcium release in the hippocampus (Hampson et al. 2009). Unfortunately, these effects have not yet translated into robust responses in clinical trials (Boyle et al. 2012; Wesensten et al. 2007), possibly due to lack of a direct measure of target engagement to inform human dosing regimens. Beyond glutamate, interactions of the mGlu5 receptor with other mechanisms postulated to play a role in sleep-wake regulation may also be important, for example Homer 1a (Ango et al. 2001; Ango et al. 2002; Maret et al. 2007) or adenosinergic transmission (Bachmann et al. 2012; Bodenmann et al. 2012; Gallopin et al. 2005; Okada et al. 2003). Finally, a recent PET imaging study in humans using the selective radioligand ^11^C-ABP688 has demonstrated that mGlu5 receptor availability is increased in several brain regions after one night of sleep deprivation (Hefti et al. 2013), which suggests that dynamic changes in mGlu5 receptor expression may play a fundamental role in sleep/wake homeostasis.

It is clear that a homeostatic process tracks the loss of sleep as a “sleep pressure” that will result in a dose-dependent compensation proportional to the debt accrued (Daan et al. 1984; Dijk et al. 1990). All treatments compared in the present study resulted in immediate attenuation of the compensatory sleep response elicited by 11 h of sleep restriction. However, the proportion of sleep pressure immediately relieved following pro-vigilant treatment was later recovered to a degree during the subsequent recording period, suggesting that requirement for homeostatic sleep was not completely alleviated by any compound. The most rapid and complete compensatory sleep response was observed for d-amphetamine, whilst both caffeine and modafinil produced equivalent proportions of NREM sleep recovery relative to the additional wakefulness gained. The greatest differential effect was observed for LSN2814617, where animals appeared to only recover 22% of lost NREM sleep versus 70% of REM sleep. This finding is interesting, as it is more typical for NREM sleep recovery to precede or at least occur concomitantly with REM sleep recovery (Berger and Oswald 1962; Borbely and Neuhaus 1979). Future work should consider how the relatively distinct effects of LSN2814617 on NREM versus REM compensatory sleep responses and performance capacity may be related.

In conclusion, the present study demonstrated that a PVT-like SRLT can be used in rats to detect behavioural impairments caused by a quantified loss of sleep. An mGlu5 PAM molecule was shown to produce marked wakefulness and an improvement in functional capacity of sleep-restricted animals, qualitatively distinct from that of amphetamine, caffeine and modafinil. The methodology and novel pharmacological effects described may offer utility for future work directed at understanding the translational correspondence of pro-vigilant drug effects between species.

## Electronic supplementary material

ESM 1 (DOCX 16 kb)

ESM 2 (XLSX 49 kb)

## References

[CR1] Ahnaou A, Langlois X, Steckler T, Bartolome-Nebreda JM, Drinkenburg WHIM (2015). Negative versus positive allosteric modulation of metabotropic glutamate receptors (mGluR5): Indices for potential pro-cognitive drug properties based on EEG network oscillations and sleep-wake organization in rats. Psychopharmacology.

[CR2] Akerstedt T, Philip P, Capelli A, Kecklund G (2011). Sleep loss and accidents—Work hours, life style, and sleep pathology. Prog Brain Res.

[CR3] Anderson C, Wales AW, Horne JA (2010). PVT lapses differ according to eyes open, closed, or looking away. Sleep.

[CR4] Ango F, Prezeau L, Muller T, Tu JC, Xiao B, Worley PF, Pin JP, Bockaert J, Fagni L (2001). Agonist-independent activation of metabotropic glutamate receptors by the intracellular protein Homer. Nature.

[CR5] Ango F, Robbe D, Tu JC, Xiao B, Worley PF, Pin JP, Bockaert J, Fagni L (2002). Homer-dependent cell surface expression of metabotropic glutamate receptor type 5 in neurons. Mol Cell Neurosci.

[CR6] Bachmann V, Klaus F, Bodenmann S, Schafer N, Brugger P, Huber S, Berger W, Landolt HP (2012). Functional ADA polymorphism increases sleep depth and reduces vigilant attention in humans. Cereb Cortex.

[CR7] Balkin TJ, Bliese PD, Belenky G, Sing H, Thorne DR, Thomas M, Redmond DP, Russo M, Wesensten NJ (2004). Comparative utility of instruments for monitoring sleepiness-related performance decrements in the operational environment. J Sleep Res.

[CR8] Basner M, Dinges DF (2011). Maximizing sensitivity of the psychomotor vigilance test (PVT) to sleep loss. Sleep.

[CR9] BERGER RJ, OSWALD I (1962). Effects of sleep deprivation on behaviour, subsequent sleep, and dreaming. J Ment Sci.

[CR10] Bird MK, Lawrence AJ (2009). The promiscuous mGlu5 receptor—a range of partners for therapeutic possibilities?. Trends Pharmacol Sci.

[CR11] Bodenmann S, Hohoff C, Freitag C, Deckert J, Retey JV, Bachmann V, Landolt HP (2012). Polymorphisms of ADORA2A modulate psychomotor vigilance and the effects of caffeine on neurobehavioural performance and sleep EEG after sleep deprivation. Br J Pharmacol.

[CR12] Borbely AA, Neuhaus HU (1979). Sleep-deprivation: Effects on sleep and EEG in the rat. J Comp Physiol.

[CR13] Boulos MI, Murray BJ (2010). Current evaluation and management of excessive daytime sleepiness. Can J Neurol Sci.

[CR14] Boyle J, Stanley N, James LM, Wright N, Johnsen S, Arbon EL, Dijk DJ (2012). Acute sleep deprivation: the effects of the AMPAKINE compound CX717 on human cognitive performance, alertness and recovery sleep. J Psychopharmacol.

[CR15] Chellappa SL, Schroder C, Cajochen C (2009). Chronobiology, excessive daytime sleepiness and depression: is there a link?. Sleep Med.

[CR16] Christie MA, McKenna JT, Connolly NP, McCarley RW, Strecker RE (2008). 24 hours of sleep deprivation in the rat increases sleepiness and decreases vigilance: Introduction of the rat-psychomotor vigilance task. J Sleep Res.

[CR17] Coenen AM, van Luijtelaar EL (1985). Stress induced by three procedures of deprivation of paradoxical sleep. Physiol Behav.

[CR18] Cordova CA, Said BO, McCarley RW, Baxter MG, Chiba AA, Strecker RE (2006). Sleep deprivation in rats produces attentional impairments on a 5-choice serial reaction time task. Sleep.

[CR19] Czeisler CA, Walsh JK, Roth T, Hughes RJ, Wright KP, Kingsbury L, Arora S, Schwartz JR, Niebler GE, Dinges DF, U.S. Modafinil in Shift Work Sleep Disorder Study Group (2005). Modafinil for excessive sleepiness associated with shift-work sleep disorder. N Engl J Med.

[CR20] Daan S, Beersma DG, Borbely AA (1984). Timing of human sleep: Recovery process gated by a circadian pacemaker. Am J Physiol.

[CR21] Deadwyler SA, Porrino L, Siegel JM, Hampson RE (2007). Systemic and nasal delivery of orexin-A (Hypocretin-1) reduces the effects of sleep deprivation on cognitive performance in nonhuman primates. J Neurosci.

[CR22] Dijk DJ, Brunner DP, Beersma DG, Borbely AA (1990). Electroencephalogram power density and slow wave sleep as a function of prior waking and circadian phase. Sleep.

[CR23] Dinges DF, Powell JW (1985). Microcomputer analyses of performance on a portable, simple visual RT task during sustained operations. Behav Res Methods Instrum Comput.

[CR24] Dinges DF, Weaver TE (2003). Effects of modafinil on sustained attention performance and quality of life in OSA patients with residual sleepiness while being treated with nCPAP. Sleep Med.

[CR25] Eagle DM, Tufft MR, Goodchild HL, Robbins TW (2007). Differential effects of modafinil and methylphenidate on stop-signal reaction time task performance in the rat, and interactions with the dopamine receptor antagonist cis-flupenthixol. Psychopharmacology (Berl).

[CR26] Gallopin T, Luppi PH, Cauli B, Urade Y, Rossier J, Hayaishi O, Lambolez B, Fort P (2005). The endogenous somnogen adenosine excites a subset of sleep-promoting neurons via A2A receptors in the ventrolateral preoptic nucleus. Neuroscience.

[CR27] Gilmour G, Broad LM, Wafford KA, Britton T, Colvin EM, Fivush A, Gastambide F, Getman B, Heinz BA, McCarthy AP, Prieto L, Shanks E, Smith JW, Taboada L, Edgar DM, Tricklebank MD (2013). In vitro characterisation of the novel positive allosteric modulators of the mGlu(5) receptor, LSN2463359 and LSN2814617, and their effects on sleep architecture and operant responding in the rat. Neuropharmacology.

[CR28] Gregory KJ, Herman EJ, Ramsey AJ, Hammond AS, Byun NE, Stauffer SR, Manka JT, Jadhav S, Bridges TM, Weaver CD, Niswender CM, Steckler T, Drinkenburg WH, Ahnaou A, Lavreysen H, Macdonald GJ, Bartolome JM, Mackie C, Hrupka BJ, Caron MG, Daigle TL, Lindsley CW, Conn PJ, Jones CK (2013). N-aryl piperazine metabotropic glutamate receptor 5 positive allosteric modulators possess efficacy in preclinical models of NMDA hypofunction and cognitive enhancement. J Pharmacol Exp Ther.

[CR29] Hampson RE, Espana RA, Rogers GA, Porrino LJ, Deadwyler SA (2009). Mechanisms underlying cognitive enhancement and reversal of cognitive deficits in nonhuman primates by the ampakine CX717. Psychopharmacology (Berl).

[CR30] Hefti K, Holst SC, Sovago J, Bachmann V, Buck A, Ametamey SM, Scheidegger M, Berthold T, Gomez-Mancilla B, Seifritz E, Landolt HP (2013). Increased metabotropic glutamate receptor subtype 5 availability in human brain after one night without sleep. Biol Psychiatry.

[CR31] Knie B, Mitra MT, Logishetty K, Chaudhuri KR (2011). Excessive daytime sleepiness in patients with Parkinson’s disease. CNS Drugs.

[CR32] Lim J, Dinges DF (2008). Sleep deprivation and vigilant attention. Ann N Y Acad Sci.

[CR33] Liu YP, Tung CS, Lin YL, Chuang CH (2011). Wake-promoting agent modafinil worsened attentional performance following REM sleep deprivation in a young-adult rat model of 5-choice serial reaction time task. Psychopharmacology (Berl).

[CR34] Ly S, Pishdari B, Lok LL, Hajos M, Kocsis B (2013). Activation of 5-HT6 receptors modulates sleep-wake activity and hippocampal theta oscillation. ACS Chem Neurosci.

[CR35] Maret S, Dorsaz S, Gurcel L, Pradervand S, Petit B, Pfister C, Hagenbuchle O, O’Hara BF, Franken P, Tafti M (2007). Homer1a is a core brain molecular correlate of sleep loss. Proc Natl Acad Sci U S A.

[CR36] Minzenberg MJ, Carter CS (2008). Modafinil: a review of neurochemical actions and effects on cognition. Neuropsychopharmacology.

[CR37] Morgan RE, Crowley JM, Smith RH, LaRoche RB, Dopheide MM (2007). Modafinil improves attention, inhibitory control, and reaction time in healthy, middle-aged rats. Pharmacol Biochem Behav.

[CR38] Okada T, Mochizuki T, Huang ZL, Eguchi N, Sugita Y, Urade Y, Hayaishi O (2003). Dominant localization of adenosine deaminase in leptomeninges and involvement of the enzyme in sleep. Biochem Biophys Res Commun.

[CR39] Porrino LJ, Daunais JB, Rogers GA, Hampson RE, Deadwyler SA (2005). Facilitation of task performance and removal of the effects of sleep deprivation by an ampakine (CX717) in nonhuman primates. PLoS Biol.

[CR40] Rechtschaffen A, Bergmann BM, Everson CA, Kushida CA, Gilliland MA (2002). Sleep deprivation in the rat: X. Integration and discussion of the findings. 1989. Sleep.

[CR41] Roth T, Roehrs TA (1996). Etiologies and sequelae of excessive daytime sleepiness. Clin Ther.

[CR42] Stocking EM, Letavic MA (2008). Histamine H3 antagonists as wake-promoting and pro-cognitive agents. Curr Top Med Chem.

[CR43] Van Dongen HP, Dinges DF, Kryger MH, Roth T, Dement WC (2000). Circadian rhythmin sleepiness, alertness and performance. Principles and practice of sleep medicine.

[CR44] Van Dongen HP, Maislin G, Mullington JM, Dinges DF (2003). The cumulative cost of additional wakefulness: Dose–response effects on neurobehavioral functions and sleep physiology from chronic sleep restriction and total sleep deprivation. Sleep.

[CR45] Waters KA, Burnham KE, O’connor D, Dawson GR, Dias R (2005). Assessment of modafinil on attentional processes in a five-choice serial reaction time test in the rat. J Psychopharmacol.

[CR46] Wesensten NJ, Reichardt RM, Balkin TJ (2007). Ampakine (CX717) effects on performance and alertness during simulated night shift work. Aviat Space Environ Med.

[CR47] Wurts SW, Edgar DM (2000). Circadian and homeostatic control of rapid eye movement (REM) sleep: Promotion of REM tendency by the suprachiasmatic nucleus. J Neurosci.

