# Supplementary material for: Distinct pro-vigilant profile induced in rats by the mGluR5 potentiator LSN2814617
Source: Psychopharmacology (Berl). 2015 Apr 24;232(21-22):3977–89. doi: 10.1007/s00213-015-3936-8 (PMC4600478; doi:10.1007/s00213-015-3936-8)
Supplement: Supplementary file 1 — (DOCX 16 kb) [file 213_2015_3936_MOESM1_ESM.docx]

**Supplemental Material for:**

**“A functionally distinct form of wake promotion is induced in rats by the mGlu5 potentiator LSN2814617” *by* Loomis S et al.**

**S1: Chronic EEG and EMG recording.** Adult male Wistar rats were anesthetized (2% isoflourane in 95/5 oxygen) and surgically prepared with a cranial implant that permitted chronic electroencephalogram (EEG) and electromyogram (EMG) recording. Body temperature and locomotor activity were monitored via a miniature transmitter (Minimitter Series PDT4000G, Philips Respironics, Bend, OR) surgically placed in the abdomen during the same anaesthetic procedure as the cranial implant. The cranial implant consisted of stainless steel screws (2 frontal [+3.9 AP from bregma, ±2.0 ML] and 2 occipital [-6.4 AP, ±5.5 ML]) for EEG recording. Two Teflon-coated stainless steel wires were positioned under the nuchal trapezoid muscles for EMG recording. All leads were soldered to a miniature connector (Microtech, Boothwyn, PA, USA) and gas sterilized with ethylene oxide prior to surgery. The implant assembly was affixed to the skull by the combination of the EEG recording screws, cyanoacrylate applied between the hermetically sealed implant connector and skull, and dental acrylic. An analgesic (buprenorphine 0.05 mg/kg SC) was administered pre-operatively and daily SC for 2 days post-surgery. Additional analgesia was provided with Metacam (meloxicam) (0.15 mg/kg PO) for 6 days post-surgery. Prophylactic antibiotics (cefalexin 20 mg/kg PO) were administered 24h and immediately prior to surgery, and daily for 7 days thereafter. At least three weeks were allowed for recovery. Implanted animals were individually housed in individually ventilated cages under standard conditions while training on the SRLT task progressed, thereafter animals were housed individually in custom-designed, sleep deprivation chambers for the duration of the experiment. Food and water were available ad libitum and the ambient temperature was 23±1°C. Each cage was provided with an infrared light source and digital video camera to allow continuous remote visual monitoring. A 24-hr light-dark cycle (LD 12:12) was maintained throughout the study using fluorescent light. Light intensity averaged 35-40 lux at mid-level inside the cage. Food and water were available ad libitum and the ambient temperature was 23±1°C. Relative humidity averaged 50% approximately.

Sleep and wakefulness were determined using *SCORE*-2004™ — a microcomputer-based sleep-wake and physiological monitoring system. Validation of the *SCORE*™ technology in rodents and utility in pre-clinical drug evaluation have been previously described (Van Gelder *et al.* 1991; Edgar *et al.* 1991, 1997; Seidel *et al.* 1995, 1998). In the present study, the system monitored amplified EEG (X10,000, bandpass 1-30 Hz [Grass Corp., Quincy, MA]; initial digitization rate 400 Hz), integrated EMG (bandpass 10-100 Hz, RMS integration), and telemetered body temperature and non-specific locomotor activity (LMA), from 16 rodents simultaneously. Arousal states were classified on-line as NREM sleep, REM sleep, wake, or theta-dominated wake every 10 seconds using EEG period and amplitude feature extraction and ranked membership algorithms. Individually taught EEG-arousal-state templates and EMG criteria differentiated states of arousal.

In addition to frequent on-line inspection of the EEG and EMG signals, quality control of the data was assured by expert analysts using a suite of programs (SCOREVIEW™, Lilly Research Laboratories) that allowed data quality of all variables to be flexibly scrutinized at the level of *(i)* individual visual examination of raw EEG and EMG signals, *(ii)* individual hourly mean timeseries, and *(iii)* group mean timeseries, using a combination of graphical and statistical assessments. Offline visual inspection of baseline signal was performed to ensure sleep-wake scoring accuracy. All off line sleep analysis was performed blind with respect to treatment group. An integrated relational database was updated with data quality control decisions for each individual treatment, and this database controlled all subsequent use of these data. Complete, digitized raw EEG and integrated EMG data were permanently archived for all treatments.
